# Risk Attitudes Affect Livestock Biosecurity Decisions With Ramifications for Disease Control in a Simulated Production System

**DOI:** 10.3389/fvets.2019.00196

**Published:** 2019-06-25

**Authors:** Gabriela Bucini, Scott C. Merrill, Eric Clark, Susan M. Moegenburg, Asim Zia, Christopher J. Koliba, Serge Wiltshire, Luke Trinity, Julia M. Smith

**Affiliations:** ^1^Department of Plant and Soil Science, University of Vermont, Burlington, VT, United States; ^2^The Vermont Complex Systems Center, University of Vermont, Burlington, VT, United States; ^3^Department of Community Development and Applied Economics, University of Vermont, Burlington, VT, United States; ^4^Department of Food Systems, University of Vermont, Burlington, VT, United States; ^5^Department of Mathematics and Statistics, University of Vermont, Burlington, VT, United States; ^6^Department of Animal and Veterinary Sciences, University of Vermont, Burlington, VT, United States

**Keywords:** agent-based models, disease transmission, biosecurity, risk attitude, human behavior, porcine epidemic diarrhea virus (PEDv), hog production

## Abstract

Hog producers' operational decisions can be informed by an awareness of risks associated with emergent and endemic diseases. Outbreaks of porcine epidemic diarrhea virus (PEDv) have been re-occurring every year since the first onset in 2013 with substantial losses across the hog production supply chain. Interestingly, a decreasing trend in PEDv incidence is visible. We assert that changes in human behaviors may underlie this trend. Disease prevention using biosecurity practices is used to minimize risk of infection but its efficacy is conditional on human behavior and risk attitude. Standard epidemiological models bring important insights into disease dynamics but have limited predictive ability. Since research shows that human behavior plays a driving role in the disease spread process, the explicit inclusion of human behavior into models adds an important dimension to understanding disease spread. Here we analyze PEDv incidence emerging from an agent-based model (ABM) that uses both epidemiological dynamics and algorithms that incorporate heterogeneous human decisions. We investigate the effects of shifting fractions of hog producers between risk tolerant and risk averse positions. These shifts affect the dynamics describing willingness to increase biosecurity as a response to disease threats and, indirectly, change infection probabilities and the resultant intensity and impact of the disease outbreak. Our ABM generates empirically verifiable patterns of PEDv transmission. Scenario results show that relatively small shifts (10% of the producer agents) toward a risk averse position can lead to a significant decrease in total incidence. For significantly steeper decreases in disease incidence, the model's hog producer population needed at least 37.5% of risk averse. Our study provides insight into the link between risk attitude, decisions related to biosecurity, and consequent spread of disease within a livestock production system. We suggest that it is possible to create positive, lasting changes in animal health by nudging the population of livestock producers toward more risk averse behaviors. We make a case for integrating social and epidemiological aspects in disease spread models to test intervention strategies intended to improve biosecurity and animal health at the system scale.

## Introduction

In recent years, the hog production industry has been subjected to incursions of both endemic and new diseases. In 2013, the first outbreak of porcine epidemic diarrhea virus (PEDv) in the U.S. shook the industry both economically and socially, and required us to rethink effective disease-prevention strategies (1, 2). PEDv is now an endemic disease and it is one of the most severe infectious diseases in the hog industry with ~80–100% morbidity and 50–90% mortality in suckling piglets (3, 4). The virus can spread via direct, indirect and possibly airborne transmission mechanisms (5–12). Direct transmission involves animal-to-animal contact while indirect transmission implies exposure to contaminated fomites. Furthermore, both animal and environment can be reservoirs of the virus for long periods, making it difficult to predict the time and place of new outbreaks (13). There is no single successful control strategy for PEDv, in part because of the complexity and large size of the swine population, but also because of poorly-understood transmission vectors, including inconsistent, and occasionally-irrational behavior by humans in the industry. Thus, one aspect of the problem has become clear: livestock disease spread is not only epidemiological but also a matter of human behavior, specifically the choices producers make to implement biosecurity protocols or not (14).

**Figure 1:**
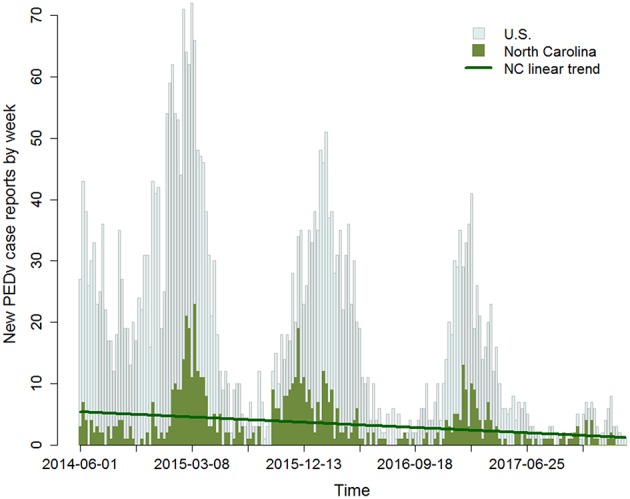
Time series of the number of confirmed new PEDv positive premises by week. Gray bars report data for the U.S. and green bars for the state of North Carolina (NC). The data are available for the period 06/01/2014 to 02/25/2018. Issued on June 5, 2014, a Federal Order required the reporting of swine enteric coronavirus diseases including PEDv (https://www.aasv.org/aasv%20website/Resources/Diseases/PorcineEpidemicDiarrhea.php). On March 6, 2018, USDA rescinded the Federal Order (https://www.aphis.usda.gov/aphis/newsroom/news/sa_by_date/sa-2018/secd-reporting). The dark green line traces the decreasing trend in incidence in NC with a slope m = −0.02. This is equivalent to an average decrease from about 20 new cases in the month June, 2014 down to eight new cases in the same month in 2017.

Observed data published by the United States Department of Agriculture (Swine Enteric Coronavirus Disease Situation report—Mar 2018^1^) show a high PEDv incidence in the winter of 2014 followed by a significant decreasing trend over each subsequent year (Figure 1). The data also exhibit seasonal cycles with winter seasons generally carrying higher PEDv incidence. While there are likely a number of factors influencing the variability in the data, we became interested in the steady decreasing trend. Since the pathways of virus transmission have stayed the same through time, why has incidence decreased? Likely, this is evidence of a change in the response to disease within the production system. We therefore investigate how shifts in human behaviors and risk mitigation strategies longitudinally affect contagion dynamics.

Biosecurity has been considered the most important prevention strategy for PEDv (14). Biosecurity practices such as disinfecting footwear, showering and wearing clean clothes before entering production premises, vehicle washing and disinfecting can be employed to mitigate PEDv transmission both within and between farms (15–17). Although producers have access to biosecurity information and implementation instructions, their risk attitude can influence the willingness to comply with biosecurity protocols (18, 19). Hereafter, we refer to this operational willingness to obey the rules as “compliance” with biosecurity protocols. Failure to comply with biosecurity practices can lead to infection, increased mortality of pigs of all ages and economic losses for the farm. A second aspect of biosecurity is the willingness by managers and owners to invest in biosecurity, for example purchasing truck-washing equipment or installing air-filtration systems. For this reason, human decision-making factors, in addition to epidemiological factors, are essential pieces needed to understand disease dynamics and their associated economic repercussions (20).

From an applied perspective, clarifying the mechanisms that link human risk attitude to biosecurity adoption and compliance will aid in understanding long-term disease risks and help to develop strategies for controlling disease incurrence (21). At the forefront of disease prevention are people involved with daily on-farm practices or decisions regarding the biosecurity standards on a farm. However, not everybody perceives disease risk in the same way (20, 22) and biosecurity practices are not applied homogenously and at the same level across farms (23, 24). Critical research on human decision-making shows that behaviors are not immutable and can be nudged toward standards that are more beneficial both for the individual and the larger community (25). In the case of disease for example, both the producer and the production system can benefit from improved disease control by shifting individual producers' behaviors toward higher biosecurity engagement (20). The integration of epidemiological and social disciplines can provide insights (26) on the effect of shifts in human behavior directed at protecting farms from disease incursions.

A useful approach for studying the mechanisms by which both epidemiological and human-behavioral factors affect disease spread is in a simulated environment where factors can be varied and tested for their effects. Epidemiological models describe the biological and environmental components of disease transmission and evolution (5, 10, 27–30) but do not address the role of human behavior in the process of spreading disease between animal production facilities (19). Melding epidemiology with human behavioral science acknowledges that people play a role in maintaining animal health and offers a potentially richer framework to understand the dynamics of disease and inform prevention strategies (18, 26).

Agent-Based Models (ABMs) have been applied to study social phenomena and analyze macroscopic patterns that emerge from the interaction of a number of agents programmed to behave according to specified rules (31, 32). ABMs are computational models that attempt to capture the behavior of autonomous agents within their environment. An ABM usually consists of: (1) Agents, which represent actors characterized by attributes and behaviors; (2) Agent relationships and functions for their interactions and; (3) an environment in which the agents are embedded and with which they can interact. Sometimes agents can be part of a population and share characteristics and/or behaviors. Agents can receive information and/or learn and therefore have adaptive capabilities. The strength of ABMs is the ability to model complex systems from the bottom up with agents that have believable and realistic behaviors (33, 34). In situations characterized by risk as in the onset of a disease outbreak, the heterogeneity of human responses can lead to complex and dynamic outcomes challenging to foresee. Therefore, modeling agents with human-like characteristics including the ability to appraise and respond to events also with non-rational behaviors, is essential for social-ecological studies (18, 35). An example of the potential of ABMs for epidemiological applications came from the Models of Infectious Disease Agent Studies (MIDAS^2^). In this collaborative effort, a set of ABMs was developed to investigate avian flu transmission incorporating epidemiological, environmental and social aspects and has been used to analyze outbreaks, model outcomes of interventions involving human behaviors and shape policies to help reduce the impact of avian influenza (34). Because ABMs allow explicit modeling of decision-making processes, interactions and networks, they represent an effective approach for simulating the system structure of the swine industry, specifically by incorporating both disease dynamics emerging from virus transmission with animal and feed movement, and human decision processes influencing biosecurity and movement interactions.

To form a better view of PEDv disease dynamics with the role of human behavior, we built an ABM at the scale of a regional hog production system. We modeled disease spread among a variety of different agents: (1) producers with different holding types (farrow-to-finish, farrow-to-wean, wean-to-feeder…), (2) feed mills, and (3) slaughter plants. The modeled hog supply chain includes both single- and multi-site production with networks of pig movement and feed deliveries. The other two main ABM components are the epidemiological and human decision-making components. The epidemiological component contains the mechanisms of PEDv transmission (direct and indirect), while the human behavioral component accounts for risk attitude and decision-making that influence biosecurity in the system. The elements of human decision-making and behavior were selected to reflect patterns observed by industry professionals to have major effects on farm biosecurity: (1) psychological distancing (36) that leads to a relaxation of compliance with biosecurity protocols as time passes without experiencing disease; (2) responsiveness to disease presence and; (3) the willingness of farm managers/owners to invest in biosecurity. The explicit inclusion of human behavior into the ABM provides a dimension for accounting for both the willingness to implement preventive biosecurity measures and to comply with them. Thus, with agent-based modeling we can represent the influence of responsiveness, heterogeneity, information exchange, psychological distancing, and interactions of humans and the environment.

This paper presents and compares the disease-spread consequences of human decision-making simulated using an ABM of a swine production system. To this end, we design agent populations with proportionately varied risk attitudes observed from an online digital field experiment. These range from risk averse strategies that allocate more preventative biosecurity during outbreaks to risk tolerant attitudes that gamble with very little biosecurity investment. As the risk attitude influences the agent behavior in our ABM, we analyzed temporal patterns of disease incidence emerging from the simulated scenarios of heterogeneity in risk attitudes within the population of producer agents.

## Methods

**Figure 2:**
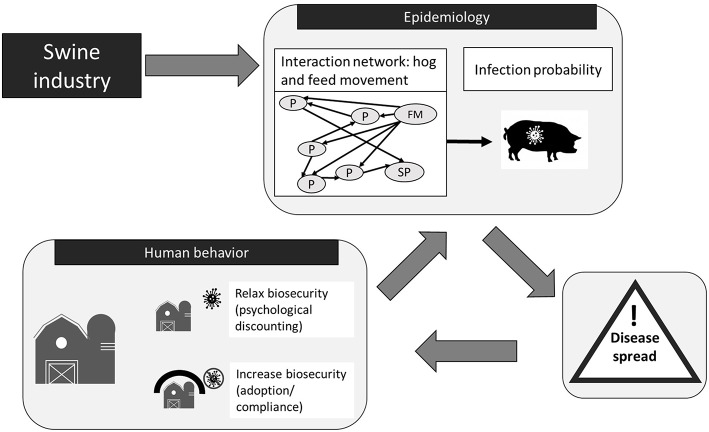
Agent-based model (ABM) process flow. It highlights the ABM's main components and processes of how the Porcine Epidemic Diarrhea virus (PEDv) can spread through the network structure of the swine industry and is influenced by human behavior. The ABM structural component mimics the swine industry with three types of agents: P, producer; FM, feed mill; and SP, slaughter plant. Agents interact via networks of hog and feed movement. The ABM epidemiological component simulates the risk of PEDv transmission associated with movement through these network connections disease spreads. Human decisions on biosecurity also influence infection risk. Disease spread depends on the probability of disease transmission on the networks and influences the biosecurity level on farms.

The agent-based model (ABM) used in this study was built off a previous ABM called “Regional U.S. Hog Production Network Biosecurity Model” (RUSHPNBM) originally created by Wiltshire et al. (37) and Wiltshire (38). The purpose of these ABMs has been the study of PEDv transmission in swine production systems. The ABMs are developed in AnyLogic^3^ software with all functions written in Java^4^. The main developments of the model for the current study include the addition of: (1) seasonal disease cycles; (2) environmental infection events simulating persistence of PEDv in the environment which allowed for reoccurrence of the disease at previously infected sites; (3) on-farm infections from visitor vehicles other than hog or feed trucks; (4) agent adaptive functionalities (e.g., human behavioral processes such as willingness to adopt biosecurity and psychological distancing); (5) risk attitude categories derived from digital field experiments and; (6) webDb database for data input and output. The model's design and implementation relevant for the current study are provided here and further details can be found in the Supplementary Material and in Wiltshire (38) and Wiltshire et al. (37). The main idea of the current ABM is to model both forward and feedback processes that describe the influence of (1) human risk attitude on biosecurity choices, (2) biosecurity on the probability of disease transmission, and (3) disease status on human decisions around biosecurity mediated by risk attitude. The ABM's process flow can be divided into a structural, an epidemiological and a human behavioral component (Figure 2), described in the following sections. The values of the model parameters are given in the (Supplementary Material).

### Structural Component: ABM Representation of the Swine Industry

The structural component simulates a hog production system with agents representing production premises, feed mills and slaughter plants. The hog production chain simulated for this study is a system mirroring the density, operation types and sizes of production units found in North Carolina with data provided by the Farm Location and Agricultural Production Simulator (FLAPS) tool which draws from the USDA Census of Agriculture and aerial images (39). Feed mills and slaughter plants were initialized at random locations with numbers obtained from public data and expert advising. Hog production in the U.S. is increasing, which has resulted in increased vertical integration. Multiple sites are used in the production flow with specialized sites for sows, weanlings, growers and finishing pigs, or any combination of these growth stages. The ABM production agents are therefore also characterized by one of six holding types (farrow-to-wean, wean-to-feeder, feeder-to-finish, farrow-to-finish, wean-to-finish, and feeder-to-finish), size (total number of animals), and number of pig batches (groups of pigs of the same age). Other structural parameters include the basic functions of the hog production system such as the process of birth and growth. Birth, growth and movements of pigs are modeled at the group level using batches of pigs of the same age. The production system of the hog industry requires transfer of hogs from one holding type to the next and in the end to the slaughter plant. For instance, in a three-site production system a pig batch moves from farrow-to-wean to wean-to-feeder to feeder-to-finish sites before finally being sent to the slaughter plant. Pig batch movement as well as feed deliveries generate heterogeneous interactions among agents and are included in the ABM using networks of transportation (Supplementary Figure 1). These networks are modeled with agents having set trading and service areas according to their industry role and characterized by neighborhood structures.

### Epidemiological Component: ABM Representation of Disease Transmission

The ABM epidemiological component is network-based and spatially explicit in that it simulates disease spread via both direct and indirect mechanisms related to the movement of animals and feed across the production network. It is coupled with a stochastic state transition model including Susceptible (S) and Infectious (I) states. Probability functions regulate the transmission of disease in single agent interactions while the network structure of animal and feed movement determine the ultimate pattern of disease spread. Each simulated agent (hog producers, slaughter plants, and feed mills) may become infected (state I) during an interaction with another agent with a probability that depends on the type of interaction, the agent's biosecurity and a seasonality factor. Specifically, each type of movement interaction is associated with an independent probability of infection calculated using a logistic function. The logistic functions describe the infection probability's dependence on the agent's biosecurity with coefficients derived from the estimates provided using expert opinion. The seasonal variability in PEDv infectivity is modeled as a sinusoidal adjustment on the logistic probability function that varies with time and ultimately generates higher infection probabilities in winter and lower in summer. Explicit representation of disease spread mechanisms and functions for our ABM are detailed in the Supplementary Material section titled The agent-based model's epidemiological sub-model.

Aside from the movement of contaminated pigs and feed, two additional sources of infection are implemented: (1) from visitors arriving at the production site and (2) from PEDv surviving in the environment within or around a production site (5, 13, 40). In our ABM, we account for the first infection source by simulating events of visitors on the production sites associated with a logistic infection probability function dependent on the producer agent's biosecurity (Supplementary Tables 1, 3). To account for the environmental infection, 0.3% of producers are randomly infected during an event scheduled once a year on a day selected from a triangular distribution defined on the range from mid-September to mid-December with mode the first week of November.

### Human Behavioral Component: ABM Representation of Biosecurity Decision-Making

We explicitly investigated the importance of capturing human behavior with interaction and feedbacks between humans and the environment. The producer agents in our ABM have adaptive capabilities and are reactive in that they do not learn but simply respond to signals from other agents and the environment. In the model, a population of veterinarian agents is encoded, each with its own network of hog producers. Within the network, the veterinarian tracks the number of hog producers affected by disease and reports it back weekly. The producer agents are encoded with a set of rules to simulate decisions to alter biosecurity at their facility in response to the disease status in their veterinarian network. Our goal was to explore the influence of reactive behaviors on biosecurity and ultimately disease incidence.

To reflect heterogeneity of human risk attitude and allow the evaluation of a variety of human behaviors, the ABM has underlying human processes with parameters for risk attitude, biosecurity investment, responsiveness to disease, and psychological distancing. In particular, the agents' risk-attitude is directly linked to their response to disease by determining the threshold number of neighboring infected production premises necessary for an agent to react and increase its biosecurity with a probability >0.9. We associate risk aversion with higher propensity to adopt biosecurity. For example, risk averse agents almost always increase biosecurity as soon as there are three production premises infected in their veterinarian network. On the opposite side of the risk spectrum, risk tolerant agents increase their biosecurity quasi certainly only when they know that there are nine or more infected production premises in their veterinarian network. In summary, the ABM agent behavior originates from a risk attitude distribution with four categories (risk averse, risk opportunists, risk neutral, and risk tolerant); four forms of disease response, one for each risk attitude category are used to simulate biosecurity response-to-disease strategies; and a utility function for psychological distancing, which simulates the waning of biosecurity compliance since an infection event. The detailed description of parameters and methods for the ABM human behavioral component are provided in the Supplementary Material section The agent-based model's human behavioral component.

### Risk-Attitude Scenarios Analysis

The goal of this study was to better understand the extent to which shifts in the composition of risk attitudes in the agent population change the incidence of PEDv outbreaks. To this end, we ran a scenario analysis where we shifted fractions of the producer population between risk tolerant and risk averse categories and then evaluated the resulting PEDv incidence. Six model scenarios were compared to a reference baseline scenario (Table 1), assigned to the case where the producer population is evenly distributed across all risk attitude categories upon model initialization. The populations of feed mill and slaughter plant agents were kept at even percentages of agents across the four risk attitude groups in all seven scenarios. The baseline scenario in particular was the reference for being the model that we calibrated against observed data. The ABM calibration was performed using AnyLogic software with the built-in genetic algorithm by matching the observed (Figure 1) and the simulated PEDv incidence. More information about the calibration methods and results can be found in the Supplementary Material, section Calibration of ABM's human behavioral component. For the six alternative scenarios (Table 1), all the model parameters were kept fixed at the calibrated values (Supplementary Table 1), while the initial proportion of population in the risk attitude groups were varied. For this analysis, the ABM was run over the time period spanning from 12/27/2009 to 02/25/2018. The first part of this period until 05/31/2014 was used to stabilize to model. The period 06/01/2014 to 02/25/2018 overlapping the observations' time series (Figure 1) produced the data for the analysis. We executed Monte Carlo experiments with 800 replicates for the seven separate scenarios and collected disease incidence data.

**Table 1:** Risk attitude scenarios.

**Scenario**	**% risk averse producers**	**% risk tolerant producers**
Baseline	25	25
12.5% averse	12.5	37.5
17.5% averse	17.5	32.5
22.5% averse	22.5	27.5
27.5% averse	27.5	22.5
32.5% averse	32.5	17.5
37.5% averse	37.5	12.5

*Each scenario represents a different initial condition in the model representing the configuration of risk attitudes in the producer agent population. Columns 2 to 3 report the relative percent of producer agents in each risk attitude group for the seven scenarios. The baseline is the scenario used to calibrate the model. The percentages of producers assigned to the risk neutral and risk averse categories are maintained fixed at 25% in all the scenarios. The increase of risk averse percentage across scenarios aligns with a larger section of the producer population adopting biosecurity with relatively higher probability as a response to disease presence*.

Statistical analyses on incidence outputs from each scenario were performed using R (41) software. We calculated summary indicators such as total incidence and linear trend coefficients, to characterize the output time series of PEDv incidence and then applied non-parametric statistical tests to compare the indicators across scenarios. Specifically we proceeded in the following ways for each summary indicator:

- Total incidence: It is defined as the sum of incidence over the simulated time period. We built distributions of total incidence from the 800 Monte Carlo replicates for each scenario. We then compared the distributions across scenarios both visually with box-plots and statistically with non-parametric tests. We applied non-parametric tests because the data did not meet either the assumption of normality (*p* > 0.0001 in Shapiro-Wilk test) or the assumption of equal variances (*p* > 0.0001 in both Brown-Forsythe test and Fligner-Killeen test). We first applied the k-sample Anderson-Darling test with all the distributions of total incidence and then compared the distributions pairwise with the two-sample Kolmogorov-Smirnov test. Box-plots were used to show the median, minimum, and maximum values and quantiles of the simulated incidence totals for each scenario.
- Linear trend coefficients: A linear regression model was fit to each of the 800 simulation runs for all scenarios and the values for the coefficients intercept and slope were collected. Box plots of intercept and slope showed the characteristics of the underlying distribution of coefficients' datasets. The non-parametric k-sample Anderson-Darling test followed by the two-sample Kolmogorov-Smirnov test were applied to compare distributions across scenarios because the data did not meet either the assumption of normality (*p*-value > 0.0001 in Shapiro-Wilk test) or the assumption of equal variances (*p*-value > 0.0001 in Brown-Forsythe test and Fligner-Killeen test). The Monte Carlo averages of both the simulated incidence and trend coefficients were calculated to display temporal patterns and trends for each scenario.

In all *post hoc* multiple pairwise comparisons with the Kolmogorov-Smirnov test, a Bonferroni adjustment was applied by testing individual hypotheses at the level α^*^ = 0.05/21 (where 21 is the number of tests).

## Results

**Figure 3:**
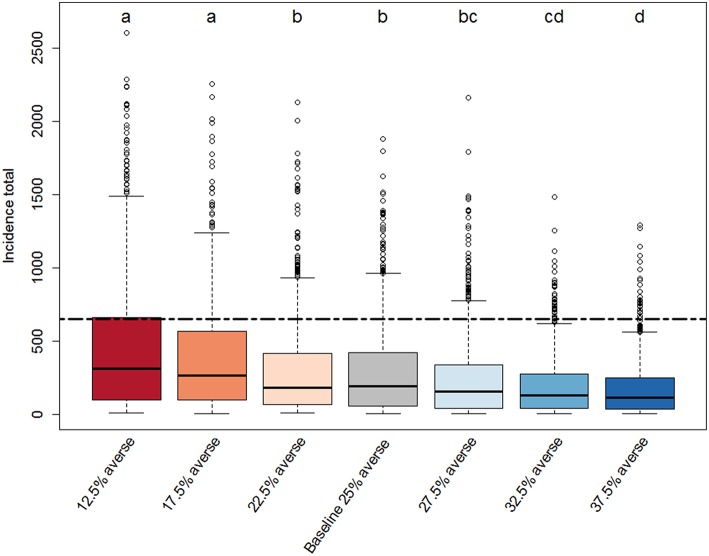
Box-plot of the distributions of total PEDv incidence (sum of new infection cases over the simulated time period) for each scenario. Each scenario represents a different distribution of risk attitudes within the population of producer agents in our ABM. The baseline-scenario population has equal proportions of producer agents in the all four groups (risk averse, risk opportunistic, risk neutral, risk tolerant). Three scenarios (12.5, 17.5, and 22.5% averse) tested the effect of reducing the number of risk averse producers by shifting a fraction (10, 30, or 50%) of producer agents from the risk averse to the risk tolerant category and are color coded with red shades. The other three scenarios (27.5, 32.5, and 37.5% averse) tested the effect of increasing the number of risk averse producers by shifting a fraction (10, 30, or 50%) of producer agents from the risk tolerant to the risk averse category and are color coded with blue shades. Each scenario distribution is drawn from a Monte Carlo experiment with 800 replicates. The compact letter display indicates significance from pairwise comparison. For the scenarios sharing a letter there is no evidence of a difference for that pair of distributions at adjusted α* = 0.002 level (Bonferroni adjustment for 21 comparisons). The black dashed line marks the total incidence in the observed data.

**Figure 4:**
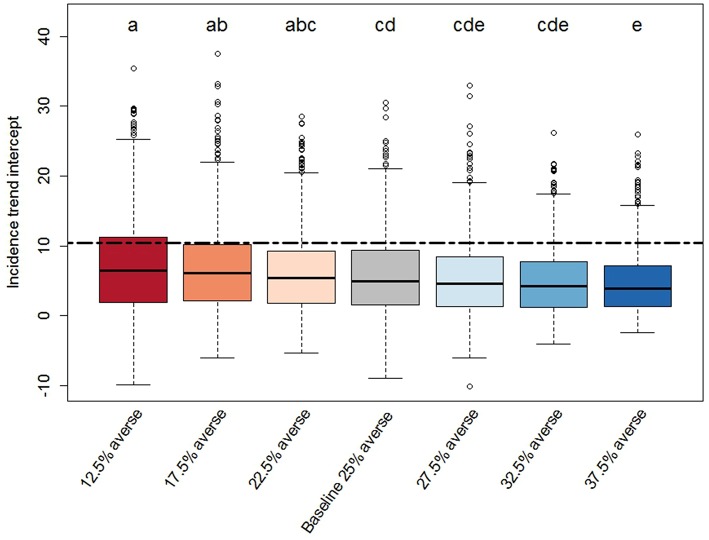
Box-plot of the intercept distributions derived from the PEDv incidence trends for each scenario. Description details as in Figure 3.

**Figure 5:**
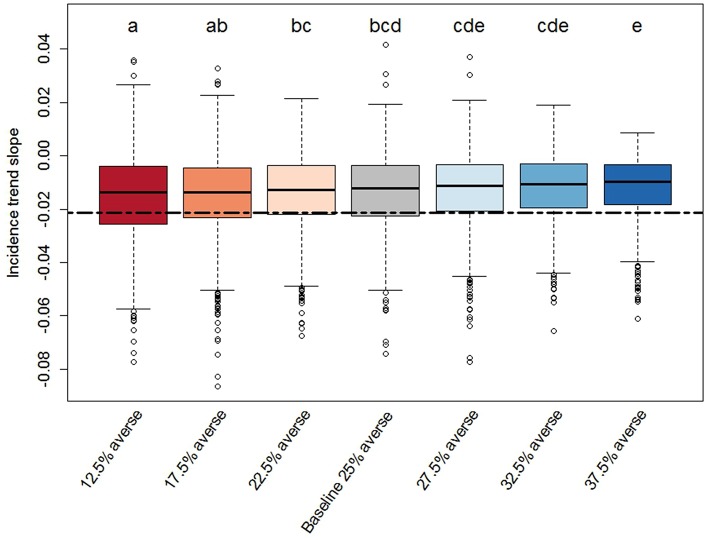
Box-plot of the slope distributions derived from the PEDv incidence trends for each scenario. Description details as in Figure 3.

The scenario analysis performed in the study addressed the sensitivity of PEDv incidence outputs given changes in the proportions of producer agents assigned to the risk averse and risk tolerant categories in the ABM. We performed statistical tests to measure the effect of seven distributions of risk attitudes (Table 1) on the spread of PEDv within the hog production system simulated in our ABM. The results of the non-parametric tests comparing the distributions of total incidence, linear trend's intercept and slope are shown by the compact letters above the box-plots in Figures 3–**5**. Box-plots are presented to show overall patterns of three indicators and visualize their distribution characteristics across scenarios. The 12.5 and 37.5% averse represent the two extreme scenarios. The baseline is the scenario with an equal percentage (25%) of producers in both the risk averse and risk tolerant categories.

### Total Incidence Indicator

The compact letters in Figure 3 show that there are some significant differences in the distributions of incidence totals across scenarios. All scenarios except for the “22.5% averse” one, which was only 10% less risk averse than the baseline scenario, have distributions significantly different from the baseline scenario. Generally, the scenarios with lower percentage of risk averse producer agents (12.5%, 17.5% averse; compact letter “a”) had more simulation runs that produced relatively high incidence totals (larger interquartile ranges) compared to the other scenarios. In contrast, scenario runs with higher proportions of risk averse producer agents (32.5 and 37.5% averse; compact letter “d”) lead to significantly different distributions of incidence totals characterized by lower medians and narrower ranges. All the scenarios appear to be right-skewed with some outlying values indicating that in all Monte Carlo experiments there were simulations where the system became very vulnerable to high PEDv infection. This is particularly evident for scenarios 12.5 and 17.5% averse. Overall, the scenarios indicate that the ABM is significantly sensitive to risk attitude shifts as small as 10% producer agents moving from being risk tolerant to being risk averse. Therefore, the total incidence indicator responds to the risk attitude distribution within the population.

The comparative box-plots provided an unexpected result when analyzed in relation to the observed total incidence (Figure 3, dashed black line). The ABM tends to underestimate the total incidence. While all scenarios produced some realizations with total incidence close to the observed one, none had the median aligned around the observed total incidence. The scenario with the most risk tolerant producers (12.5% averse) provided the highest number of simulation runs close to the observations in terms of total incidence. These results may suggest that we need to adopt a baseline model that is calibrated on an initial population of producers with relatively higher percentage of risk tolerant. Alternatively, the current baseline model could be correct and the observed data could represent a rare case that happened to be actualized in reality. Only independent data on risk attitude collected from a sample of producers can help answer this question.

### Trend Intercept and Slope Indicators

The linear regressions fit on the incidence data in relations to time provided significant trends (Table 2). The R-squared of the linear models are <0.2 ± 0.14 reflecting the high variability in the data mostly due to the seasonal cycles. Even with the high variability, the data provide significant trends and information about disease incidence change with time. The median *p*-values show significant trend for most of the simulation runs. The average *p*-values further indicate the presence of outlier regressions with non-significant trends. Overall, the data support the existence of significant changes in the incidence with time.

**Table 2:** Regression model fitness indicators.

**Scenario**	**Model R-squared**			**Model** ***p*****-values**		
	**Mean**	**Std. dev**.	**Median**	**Mean**	**Std. dev**.	**Median**
Baseline	0.2	0.14	0.18	0.03	0.12	6.40E-10
12.5% averse	0.18	0.14	0.15	0.04	0.13	1.87E-08
17.5% averse	0.19	0.14	0.18	0.03	0.13	4.14E-10
22.5% averse	0.2	0.14	0.19	0.04	0.15	1.85E-10
27.5% averse	0.2	0.14	0.18	0.02	0.11	3.65E-10
32.5% averse	0.2	0.14	0.18	0.03	0.12	8.75E-10
37.5% averse	0.2	0.14	0.19	0.03	0.13	2.34E-10

*Summary statistics of p-values and R-squares of the linear regression models (trends) of disease incidence vs. time. For each scenario, 800 regression models were fit*.

We found significant effects of risk attitude shifts in the coefficients describing the linear trends of incidence through time (Figures 4, 5). In general, the two extreme scenarios (12.5 and 37.5% averse) showed significantly different distributions compared to the baseline scenario. For example, a shift of risk averse agents from 25% (baseline) to 37.5% (more risk averse population) results in a steeper median trend (20% more negative), in other words, disease spread decreases faster. When we look at intercepts, an initial producer population with 37.5% risk averse agents created a situation where the PEDv virus had less infectivity since the simulation start with a median intercept of disease incidence 22% smaller than the intercept of the baseline scenario. We could not claim statistical support for a difference in the distribution of intercepts and slopes between the baseline scenario and the close scenarios (17.5, 22.5, 27.5, and 32.5% averse) except for the case of the intercept distribution for the 17.5% averse scenario (same compact letters in Figure 4).

In all scenarios, more than 75% of the simulation runs provided a linear trend with negative slope and positive intercept capturing the same linear trend shown in the observed historical PEDv incidence. This means that most of the simulations reproduced a situation where the disease incidence was higher at start (June 2014) and decreased with time. Fewer runs (<25%) in each scenario showed instead a positive trend indicating some model realizations in which the PEDv outbreak led to a growing incidence through time. These positive-trend cases emerge in the stochastic approach of Monte Carlo experiments where, by the law of large numbers (of simulations) more rare outcomes may also be realized. These cases accentuate and call the attention to the stochastic nature of disease spread dynamics indicating that there can be unexpected outcomes of disease spread even when the system is calibrated to contain and reduce infection.

**Figure 6:**
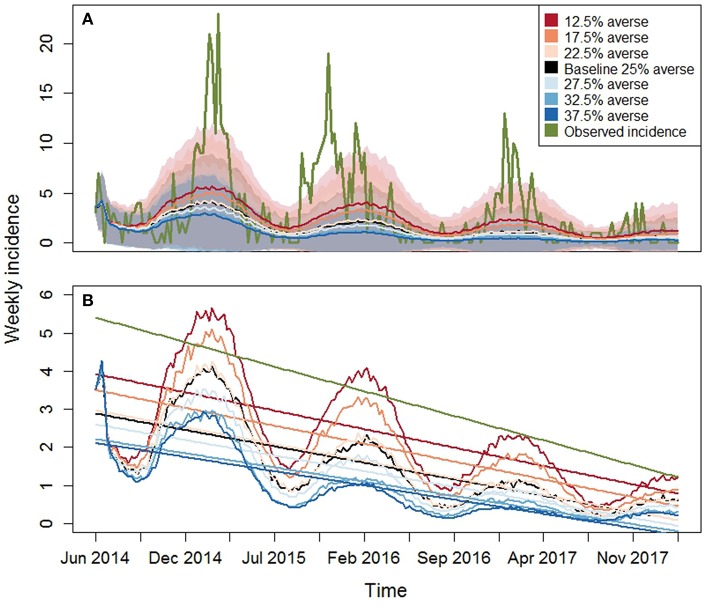
Model results for PEDv incidence for the seven risk attitude scenarios (Table 1). Observed PEDv Incidence and its linear trend are overlaid in green. **(A)** Time series of averaged simulated PEDv incidence (lines) and one-standard-deviation bands derived from the 800 Monte Carlo runs for each scenario. **(B)** Zoom on simulated outputs with overlaid trends obtained from averaged linear regressions on each simulation run. The green line represents the linear trend of the observed data. The other colors are the same as described in the legend of the top panel.

The observed intercept and slope falls either outside or at the edge of the inter-quartile range for all the scenarios indicating that most of the model simulations realized a weaker decreasing trend compared to the observed one. This means that the ABM parameterization tends to create dynamics of disease spread with overall lower incidence across time than what occurred in reality. The graphs in Figure 6 display the time series of PEDv incidence for the observation data and for the scenario averages, calculated across the 800 Monte Carlo runs, along with their trends. Our outputs demonstrate that the mechanisms and parameterization of the ABM are capable of reproducing decreasing PEDv incidence through time thanks to the dynamics of human behavior where agents could respond to PEDv presence by increasing biosecurity. In other words, the human behavioral assumptions built into our ABM influencing biosecurity and disease transmission probabilities, allowed the realizations of negative incidence trends. Furthermore, the higher propensity to increase biosecurity assigned to risk averse agents did result in lower incidence when there was a sufficient number of risk averse agents in the population. Despite the fact that most of the simulations missed the observed initial high peak of incidence, the shaded areas displaying the averaged Monte Carlo outputs plus and minus one standard deviation demonstrate that the ABM did realize disease outbreaks with high peaks of incidence.

## Discussion

The epidemiological data on PEDv available for the period of June 1st, 2014 to February 25th, 2018 shows a decreasing trend in PEDv incidence. Because the characteristic pathways of infection of the virus have not changed over time, we deduced that something has been changing in the hog production system that has improved the control of the virus. Both the literature and collaborating stakeholders refer to human behaviors with respect to both in compliance with and investment in biosecurity as critical for disease-protection management. This implies a key role of humans in the processes of controlling virus transmission. To better understand how changes in behavioral patterns could reflect changes in PEDv incidence, we developed an agent-based model (ABM) able to examine the role of human risk attitude to PEDv incidence within a simulated production system. Our model outputs reproduced a significant decrease in PEDv incidence through time. An important finding from our scenario analysis was that the average decreasing trend is significantly affected by the model's initial state, defining the proportion of the producer agents assigned to two risk categories, risk averse and risk tolerant. An increase as small as 10% more risk averse producer agents resulted in a 19% decrease in the median total PEDv incidence, which is equivalent to 36 fewer PEDv cases over the course of the analysis period (~4 years). To observe a significantly steeper decrease in incidence requires that more than 37.5% of the population be in the risk averse category. The implication is that biosecurity adoption and influencing factors of adoption (for example risk attitude) are a critical consideration when creating strategic plans or policies for disease control. Our modeling analysis reinforces the message found not only in field-specific papers but also in general papers such as in (42) who calls for developing more effective approaches for integrating social dynamics of epidemics to build more realistic models.

PEDv incidence data are highly variable and reflect the complex social-ecological structure of the swine industry. While the Monte Carlo results capture much of the system variability, different parameter sets appear to more closely align with the observed PEDv data (Figures 3–6), i.e., the initial conditions allowed us to calculate the fraction of simulation runs whose patterns are statistically close to the recorded incidence patterns. An interesting finding is that a producer-agent population with only 12.5% agents in the risk averse category resulted in statistical indicators where the median is closer to the observed value. In considering potential adjustments for our model, this result suggests to use the risk-attitude distribution from 12.5% averse scenario as a model set-up for realizations closer to the observed PEDv pattern.

An aspect of complexity present in the observed data is their variability at several time scales including weekly, seasonal, and inter-annual variability. The inter-annual variability for example is visible in the timing of the observed incidence peaks (Figure 6 top panel, example: the 2015–2016 winter peak occurred earlier than in 2014–2015). Our ABM uses a sinusoidal function calibrated to peak in January and therefore produces incidence oscillations that are more regular with time. A variety of reasons can be postulated to explain the complex variability in the observations including weather variability, changes in production components and/or routes and stochastic factors affecting disease spread. Our model simulates a closed production system where all the hog and feed movements are bounded within the region. Even if designed around the North Carolina configuration of production premises, the model does not include the complex network system that extends beyond the state boundary into other U.S. states. These out-of-state movements add potential for disease transmission and may contribute to the higher observed incidence compared to the averaged simulated one.

Human behavior and decision making represent a challenge in the animal production industry because of their complex interconnectedness with protection from disease (18, 19, 23, 43, 44). By weaving human behavioral components into epidemiological processes, our ABM is a unique tool for evaluating the effects and efficacy of disease control strategies compared to more traditional epidemiological models that lack social dynamics. Our ABM was equipped with two behavioral processes that act in opposition: (1) responsiveness to regional disease incidence with consequent increase in biosecurity and (2) psychological distancing with consequent decrease in biosecurity as time increases since an infection. Model calibration provided the appropriate tension between the two processes to match the observed decreasing trend in PEDv incidence. With these two behavioral processes we were able to capture important features of the PEDv dynamics as shown in our results. We recognize however that there is a variety of interplaying socio-psychological factors that influence decisions, as skillfully illustrated by Mankad (18). Yet our ABM is a simplified but progressive effort toward more realistic representation of epidemics.

PEDv is highly contagious and lethal in piglets that has resulted in substantial losses for the North America's swine industry. All industry actors are aware of the devastating consequences of disease incursion. The regular reemergence of PEDv indicates that there is still work to do on the epidemiology and microbiology of the virus but also on the role of humans, which necessitates the investigation of practices carried out in the industry and behaviors that allow the virus to survive and become active. Intensive research efforts in the past 5 years have brought new information about the viability of the virus (5, 7, 12, 30, 40, 45–47), and vaccines have been researched in various countries around the world. Vaccine efficacy has shown to be low (48) although a recent study had promising results with a new vaccine that was immunogenic and effective in growing pigs (49). Prevention of the virus therefore relies on good biosecurity practices with active participation of producers and all industry stakeholders in this complex supply chain network. Crucial for biosecurity to work is the proper training of staff and a culture of compliance with the protocols.

Human risk attitude is a driver when examining the role of human behavior as a factor in disease transmission. Our study suggests that shifting producer attitudes toward risk aversion is beneficial for the whole production system because it will result in reduced disease incidence. In balancing cost and benefits of biosecurity, our modeling outputs show that an engaged effort from the population of producers toward more risk averse, biosecure behaviors (e.g., readiness to enhance biosecurity and limiting psychological distancing) is effective in the control and reduction of PEDv spread. Our study points at the substantial opportunity provided by shifting behavior; however, from a production system perspective, altering a substantial proportion of a population's behavior represents a significant challenge. Yet, significant progress has been shown in other industries, for example when we alter choice architecture and provide behavioral nudges (25, 50).

Here we demonstrated the need to better understand the cognitive processes underlying decision-making about biosecurity, and highlight possible realizations of the impact of changing behavior on the spread of disease in the swine industry. However, in this research we coded biosecurity investment decisions based on the risk of acquiring a disease. Obviously, disease risk is an important factor when considering biosecurity, but it is not the only factor. A complex array of factors exists that influence biosecurity decisions that differ by individual and further depend upon the objectives of the organization, regional policies, logistical factors, and the array of behaviors by other actors in the swine network (e.g., feed mills, truck drivers, veterinarians, slaughter plants, processors, auction houses, etc.). Yet, research has shown that risk attitude can be an important decision-making factor. Like all models, our “model is wrong, but hopefully it is useful” (attributed to George Box 1976) because it provides a quantitative approximation for how human behavior and decisions can influence the spread of disease.

## Conclusion

The onset of PEDv in the U.S. hog industry was a singular experience for all stakeholders because of its high infectivity and rapid spread. Data show however that in 4 years, PEDv's potent spread appeared constrained with overall incidence reduced. Social dimensions can play a significant role in the biosecurity decisions of swine producers. We geared our epidemiological model with human behavioral processes connected to biosecurity and disease, and demonstrated the opportunity and impact associated with changing biosecurity behavior on PEDv incidence or, with a more positive spin, a healthier animal production systems. If on one side, targeted interventions to critical nodes of a production system may prove important to inhibit disease “super-spreaders,” on the other, our study shows that shifts in the overall industry toward a more risk averse culture can yield more biosecure facilities along with consistent and long-term industry-wide protection from disease.

## Author Contributions

GB, SW, EC, and SCM assisted with design and conceptualization of the agent-based model. EC helped with data collection. GB, SCM, EC, and SMM worked on data analysis. Project funding was generated with the help of JS, SCM, CK, and AZ. Experiments were conducted by GB and EC. Software development was primarily led by GB, SW, and EC. Initial manuscript drafts were created by GB. Subsequent manuscript editing was completed by all authors.

### Conflict of Interest Statement

The authors declare that the research was conducted in the absence of any commercial or financial relationships that could be construed as a potential conflict of interest.

## Footnotes

^1^http://www.aphis.usda.gov/animal-health/secd

^2^https://www.epimodels.org/drupal-new/

^3^https://www.anylogic.com

^4^https://www.oracle.com/technetwork/java/index.html
