# Supplementary material for: Risk Attitudes Affect Livestock Biosecurity Decisions With Ramifications for Disease Control in a Simulated Production System
Source: Front Vet Sci. 2019 Jun 25;6:196. doi: 10.3389/fvets.2019.00196 (PMC6604760; doi:10.3389/fvets.2019.00196)
Supplement: Supplementary file 1 [file Data_Sheet_1.pdf]

Supplementary Material

1 Figure 1S

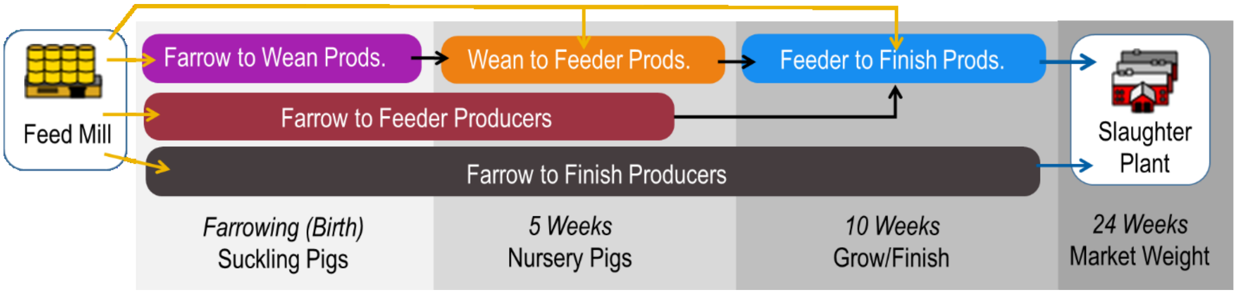

Supplementary Figure 1. Schematic showing the agent interaction flow underlying the ABM spatial network structure. Figure adapted from Figure 1. in (Wiltshire et al., 2019; Wiltshire, 2018). Pigs are transferred to the appropriate agent (another producer agent or the slaughter plant) once they reach the designated age as indicated in the bottom section of the figure. Feed mill agents deliver feed periodically to all types of producer agents.

2 Agent-based model parameters

Supplementary Table 1. Parameters describing the hog production network structure, the epidemiological sub-model (except for infection probabilities described in the Supplementary Table 3) and the human behavioral components of the ABM. These values were kept fixed for the scenario experiments.

Parameter	Value
Network Makeup	
Network connections structure	Nearest neighbor
Number of producers	2217
Number of slaughter plants	24

Number of feed mills	50
Number of veterinarians	44
Number of producers per veterinarian	50
Epidemiological Characteristics	
Number of producers initially infected	3
Number of feed mills initially infected	1
Suckling pig mortality rate	0.98
Nursery pig mortality rate	0.75
Grow/finish hog mortality rate	0.25
Length of producer infection (days)	50
Length of feed mill contamination (days)	20
Length of slaughter plant contamination (days)	60
Percent producers infected with annual environmental infection	0.3
Visitor frequency (times per week)	1.5
Farrowing	
Frequency of farrowing (days)	30
Minimum farrowing quantity as a proportion of producer capacity	0.25
Producer to Producer Pig Transfers	
Minimum transfer quantity as a proportion of transferee capacity	0.01
Feed Delivery	
Frequency of feed distribution trips (days)	2
Percent of producers in feed mill service area visited per trip	1.125
Human behavior	
Biosecurity increase	1.4
Psychological distancing rate of infected producers	0
Psychological distancing rate of clean producers	0.008
Relative shift between curves of probability of biosecurity increase across risk attitude groups	0.09

3 The agent-based model's epidemiological sub-model

PEDv can be transferred from one ABM agent to another on the network connections used for animal and feed movement. Associated with each movement there is a probability to transmit or contract infection dependent on the type of movement and the biosecurity of the interacting agents. The agent interactions are mediated by a theoretical truck, both in the case of feed deliveries, and of pig movements among producers or between a producer and a slaughter plant. The probability of infection for each type of interaction is calculated using an independent logistic equation multiplied by a seasonal adjustment. We model the logistic functions to reflect the fact that agents are less likely to become infected when more biosecurity measures are taken to prevent virus incursion. Because PEDv is a seasonal virus with highest infection risk in winter months, we add a seasonal adjustment that modulates the infection probability with time. The probability of infection is given by the following function (Supplementary Figure 2):

$$p_{infection} = adj_{season} \cdot p_{logistic}$$

Where the logistic probability $p_{logistic}$ and the seasonal adjustment adj_{season} are defined by:

- The logistic equation:

$$p_{logistic} = (p_{max} - p_{min}) \cdot \frac{1}{1 + e^{m \cdot (bs - bs_0)}} + p_{min}$$

- And the sinusoidal function:

$$adj_{season} = (1 - min_{adj}) \cdot \frac{1}{2}(1 + \cos(2\pi \cdot \frac{d - d_{peak}}{366})) + min_{adj}$$

and the parameters are described in Supplementary Table 2. For parsimony in the model's parameter values, we set p_{min}=0.05, m=1.3, and bs_0 = 4 for all agent interactions. We then estimated the logistic's p_{max} to fit the probability estimates provided by field experts for different types of interactions between hog production premises, feed mills and slaughter plants with a medium biosecurity during the high infection months of winter (Supplementary Table 3). The seasonality adjustment function adj_{season} oscillates between a maximum $max_{adj} = 1$ reached at day $d_{peak} = 30$ (January 30th) and a minimum $min_{adj} = 0.3$ in the summer.

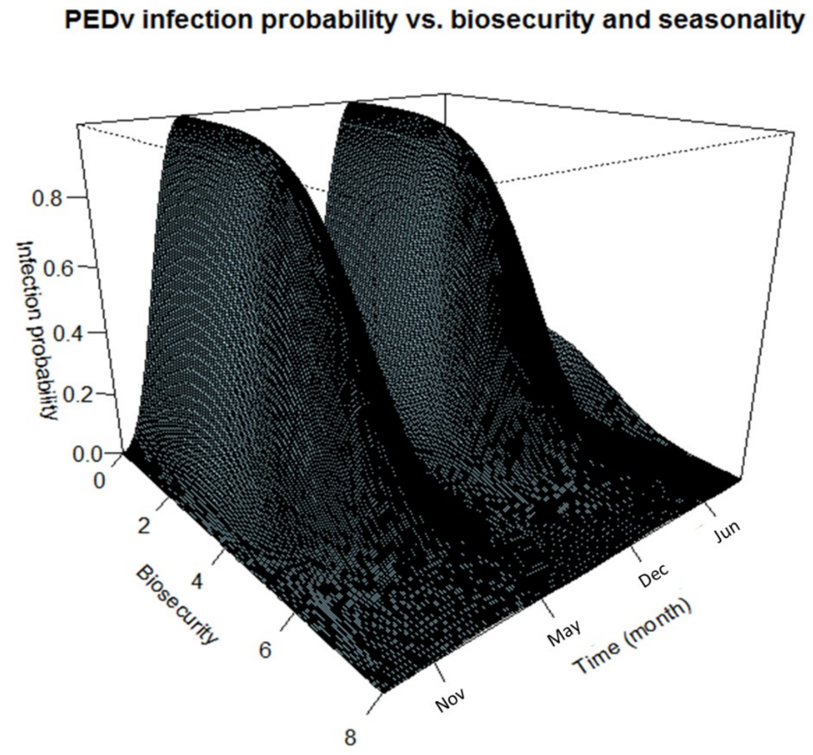

Supplementary Figure 2. Shape of the PEDv infection probability function used in the ABM. The probability of infection increases with decreasing values of biosecurity. The sinusoidal variability through time accounts for PEDv seasonality with maxima in winter (January) and minima in summer (June).

Supplementary Table 2. Parameters of the probability of infection function including both the logistic and the sinusoidal forms.

**Symbol**	**Infection probability function's term**	**Definition**	**Description**
bs		Input biosecurity	Continuous variable describing an agent's biosecurity over the range [0, 8].
p_{max}	$p_{logistic}$	Carrying capacity	$p_{max} \leq 1$<br>A set maximum value for the probability (i.e. what happens at high/infinite biosecurity).
p_{min}	$p_{logistic}$	Minimum value	Probability value at biosecurity = 0
m	$p_{logistic}$	Slope of the curve	Parameter is related to the steepness of the curve at point x0. Note that m < 0 to have a Z-shaped function
bs_0	$p_{logistic}$	Point of inflection	The point on the Z-shaped curve halfway between *minf* and *K* (i.e. the center of the logistic curve).
d	adj_{season}	Input day of the year	Current day of the year [1, 365] or [1, 366] for leap years
d_{peak}	adj_{season}	Day with highest infection (peak)	Day of the year at which the adjustment is equal to 1.
min_{adj}	adj_{season}	Scaling parameter	Parameter to keep the seasonal adjustment bounded within [min_{adj},1]

Supplementary Table 3. Logistic probabilities used in the ABM to model the epidemiological spread of PEDv associated with movement (column 1). The table breaks down the probabilities by movement/interaction type (column 1 and 2). A logistic function with four parameters {p_{max}, p_{min}, m, bs_0} models the probability of PEDv transmission as a function of the biosecurity of the agent involved in the interaction (column 3). The logistic function with parameters p_{max} (column 4), p_{min}=0.05, m=1.3, and bs_0 = 4 provides the probability of infection when biosecurity=4 on January 30 for all the probability functions. Only p_{min} for the probability function for the infection from visitors at the producers sites (last raw) is different with a value p_{min}=0.005.

**Infection probability**	**Movement/ interaction type**	**Agent's biosecurity dependence**	**Infection probability parameter p_{max}**
Prob. producer will become infected if returning pig truck is contaminated	Pig transfer between premises	Producer receiving pig truck	0.35
Prob. producer will become infected if feed truck is contaminated	Feed delivery	Producer receiving feed truck	0.8
Prob. feed truck will become contaminated if producer is infected	Feed delivery	Producer receiving feed truck	0.15
Prob. pig truck will become contaminated if producer is infected	Pig transfer between premises	Producer sending pig truck	0.4
Prob. feed mill will become infected if returning feed truck is contaminated	Feed delivery	Feed mill receiving feed truck	0.25
Prob. feed truck will become contaminated if feed mill is infected	Feed delivery	Feed mill sending feed truck	0.99
Prob. slaughter plant receiving area will become infected if pig batch is infected	Pig transfer to slaughter plant	Slaughter plant receiving pig truck	0.99

Prob. pig truck will become contaminated if receiving area is infected	Pig transfer to slaughter plant	Slaughter plant receiving pig truck	0.25
Prob. producer will become infected if visitor truck if is infected	Random event without movement of pigs or feed	Producer receiving visitor truck	0.008

4 The agent-based model's human behavioral component

Agents in our model are distinguished by several attributes, some of which describe and drive human decision-making and behavior in relation to biosecurity, namely, risk attitude, psychological distancing, and biosecurity increase and disease-response parameters for the function modeling the probability of deciding to increase biosecurity. The combination of risk attitudes and biosecurity behaviors builds the unique identity of the agents. The producer agents in particular have the ability to respond to their environment represented in our ABM by the epidemiological conditions. The relevant information is sent to producer agents in messages from their veterinarian agent. The following paragraphs describe the human behavioral features of our ABM.

Risk attitude and biosecurity

In the setup stage, the model distributes the agents of each population (producers, feed mills and slaughter plants) among four risk attitude categories: risk tolerant, risk neutral, risk opportunist and risk averse. These four risk categories reflect risk attitudes observed from an online digital field experiment (data not shown). The participants' strategies ranged from *risk averse* strategies that allocated more preventative biosecurity during outbreaks to *risk-tolerant* attitudes that gamble with very little biosecurity investment. A third observed risk strategy category was noted in the digital field-experiment data and delineated by individuals who invested resources in biosecurity when there was a high risk of disease and invested little to none in biosecurity during low risk scenarios. We refer to this group as *opportunistic*, in contrast to a fourth category, *risk neutral,* categorized with participants that did not adjust their biosecurity investment behavior with regard to observed risk of infection. In the agent-based model, the risk attitude assignment is done by way of a risk-attitude distribution for each agent population that randomly assigns one of the four categories to each agent. The relative proportion of each risk attitude category in the distribution is set by the user. In this way, it is possible to create agent populations with different risk attitude characteristics. Concretely, we used this feature in our study to create scenarios where we varied the relative proportions of risk tolerant, risk neutral, risk opportunist and risk averse agents in the population of producers and tested the effect of risk attitude shifts in the disease incidence outcome.

An important aspect of our ABM is the link between the agent's risk attitude and biosecurity. We built this link by use of distributions associated with each risk category that characterize the biosecurity values for the specific category. While this feature allows characterization of the initial biosecurity landscape based on risk attitudes, for the study presented in this paper we assume the same distribution across all risk categories. Specifically, we assume that the initial (i.e. before the onset of PEDv) agent's biosecurity is drawn from the same distribution regardless of their risk-attitude category. However, after the first simulated PEDv outbreak, the agents' risk attitude drives the decisions on whether or not to increase biosecurity and therefore modulates the biosecurity landscape in the production system affecting disease transmission probabilities.

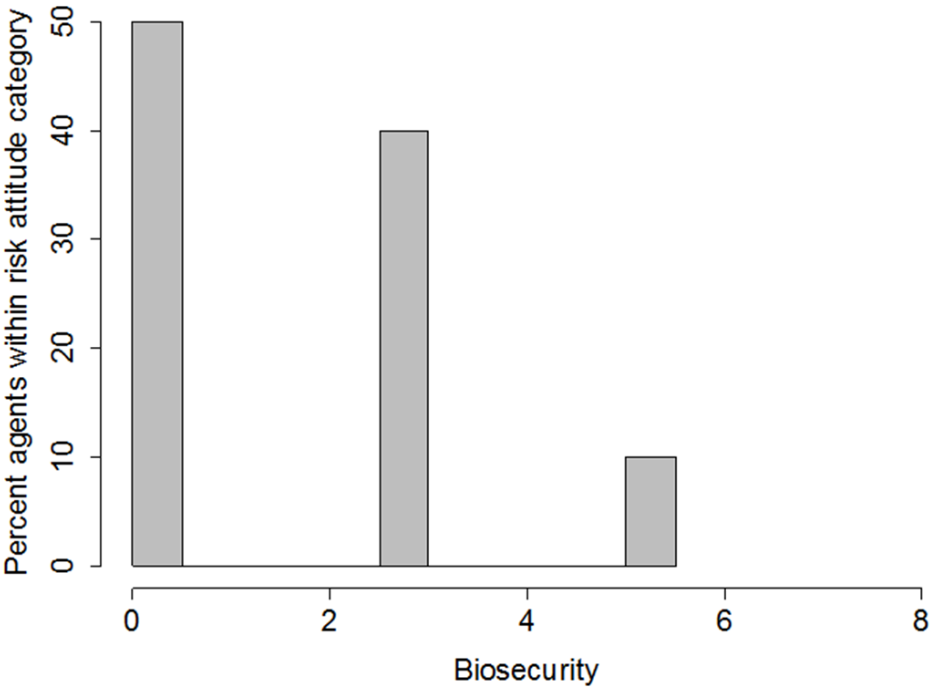

Supplementary Figure 3. Distribution of initial biosecurity values for agents in all the risk-attitude categories. The biosecurity in the ABM can vary continuously on a range [0, 8]. The distribution in the figure represents the simulated case where initially 50% of the agents have no biosecurity (biosecurity=0), 40% have low biosecurity (biosecurity=2.7), 10% have medium biosecurity (biosecurity=5.3) and 0% have high biosecurity (biosecurity=8).

Responsiveness and biosecurity increase

In our ABM, the farm agent's intention to invest in biosecurity or not is influenced by two factors: the agent's risk attitude and the information on disease available to the agents. Each producer agent is connected with a veterinarian agent that collects information about disease occurrence in its farm service network and reports the number of infected farms weekly across the network. In our model, the probability that a farm agent increases its biosecurity level upon receiving the veterinarian's message is described by a logistic function dependent on the number of farms infected in the agent's environment (Supplementary Figure 4):

$$p_{logistic} = (p_{max} - p_{min}) \cdot \frac{1}{1 + e^{m \cdot (NI - rs * NI_0)}} + p_{min}$$

Where *NI* is the number of infected producers in the veterinarian's network where the agent belongs. The parameters p_{max}, p_{min} and m are as described in Supplementary Table 2 and the parameter NI_0 is the number of infected producers at the inflection point. The parameter values of the logistic function vary for each of the four possible risk attitude categories to which each farm agent is assigned (Supplementary Table 4). The *rs* parameter controls the relative distance among the probability curves (NI_0 values) describing the biosecurity increase for four risk categories. For example, agents in the risk averse category increase biosecurity more promptly in response to disease presence than agents in the risk tolerant category, who instead probabilistically start to increase biosecurity at higher infection numbers (Supplementary Figure 4). In the model, the intention probability curves are converted to behavior by drawing a random value from a standard uniform distribution. If the random number is smaller than the probability of to increase biosecurity, the producer agent increases its biosecurity level by a fixed quantity; otherwise, the agent maintains its current biosecurity level.

Supplementary Table 4. Parameters of the biosecurity increase logistic functions for each of the four risk categories (risk averse, risk opportunist, risk neutral, risk tolerant). These values were estimated with a calibration experiment matching observed and simulated PEDv incidence and kept fixed for the scenario experiment presented in this study.

**Logistic probability function**	**Risk attitude categories**			
**Parameter**	**Risk averse**	**Risk opportunist**	**Risk neutral**	**Risk tolerant**
p_{max}	1	1	1	1
p_{min}	0	0	0	0
m	-0.95	-0.95	-0.8	-0.8
NI_0 with *rs*=0.09	0.18	1.53	2.88	4.23

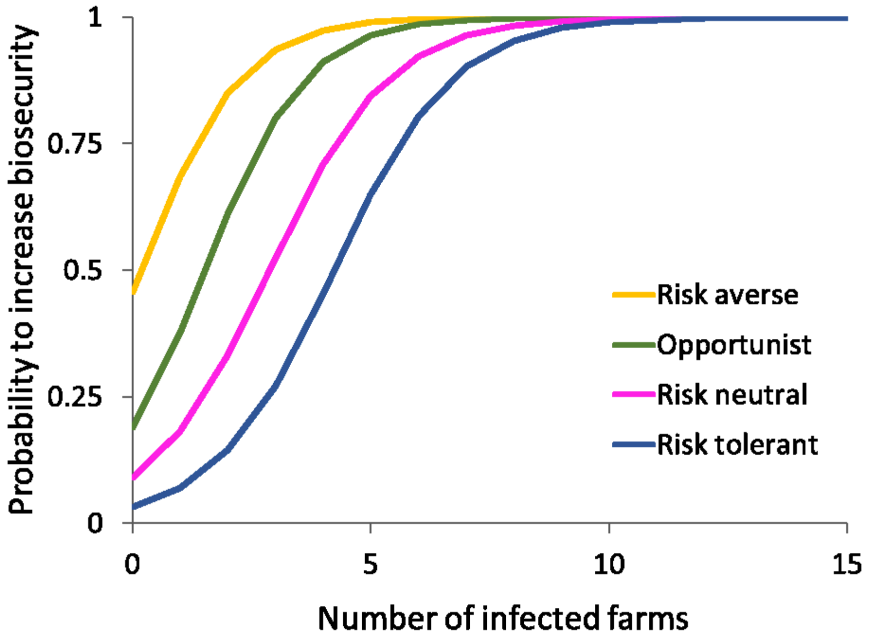

Supplementary Figure 4. Biosecurity increase logistic functions. The agent's probability to increase (or not) biosecurity is associated with the information about disease presence measured by the number of infected farms in the agent's veterinarian network. Once a week, the veterinarian agent communicates the disease information across its network of producers. In each communication event, a producer responds (or not) with an increase of biosecurity according to the logistic probability designed for the producer's risk attitude. The risk attitude influences the delay of response to the disease information as shown by the colored coded lines. With a relative shift parameter *rs*=0.09, risk averse producers have 50% chance of increasing biosecurity even when there is no infection in their veterinarian's network and their response reaches 99% at 5 infected farms. The 99% probability trigger for risk tolerant is 10 infected farms.

<u>Psychological discounting and relaxation of biosecurity compliance</u>

A relevant element of this model is psychological discounting, whereby the potential of maintaining an adopted biosecurity level is discounted through time with decreasing biosecurity compliance. The approach assumes that as time passes without infections on the agent's farm, the perception of risk of infection decreases. The functional form that we adopted for such discounting is linear with time:

$$B_t = (1 - D) \cdot B_{t-1}$$

With B_t denoting the biosecurity level of a producer agent at time t and D the fixed discounting rate. The discounting process is only active during times when the agent is the susceptible state to reflect the situation in which the absence of disease leads to a relaxation in biosecurity compliance. Instead, during time when the agent is infected, we assumed high compliance with biosecurity protocols and the value of the parameter D in the model is set to 0.

5 Calibration of ABM's human behavioral component

The goal of the calibration was to estimate human behavioral parameters that are difficult to identify by direct evidence. The incidence records provided important reference patterns to be matched by our ABM. This required applying an optimization algorithm to the following three model parameters: response delay for biosecurity adoption, increase of biosecurity and psychological discounting. During the optimization, the other model parameters related to the system structure and epidemiological mechanisms were not varied and their values were set to values estimated from expert opinion. The ABM was run over the simulating period spanning from 12/27/2009 to 02/25/2018. The period until 05/31/2014 was used to reach stabilization of the model and both the observed and simulated datasets were set to 0 incidence values. On 06/01/2014 coinciding with the first observed PEDv value, the model starts a disease outbreak to randomly infect three producer agents as indicated by the first observed incidence value. From this date the actual model calibration takes place.

The calibration experiment had an objective function (OF) set to mathematically minimize the discrepancy between the observed and simulated response variable (weekly PEDv incidence). The OF values are calculated for each model iteration by the method of least squares. Specifically, each OF value is the square root of the average of square of difference between linearly interpolated data sets (observed and simulated output). The integration range is the intersection of argument ranges of these data sets. Prior to calibration, we experimented with parameter values to visually eliminate implausible ranges. We did not impose any relation or assumed correlation between parameters. The calibration was performed for over 15000 simulations by using the OptQuest optimization package supported in the AnyLogic software. OptQuest is an optimizer that uses scatter search. We ran the calibration experiment twice for different value ranges of the three parameters. In the first calibration, we started with broad ranges and narrowed them to more suitable ranges to match the records. In the second simulation, we refined the optimization search with smaller value steps over the narrow ranges (Supplementary Table 4).

Lacking empirical information on risk attitude, we made the assumption of populations with equal proportions (25%) of agents in each of the four risk attitude categories. Within each risk category, the biosecurity configuration at model startup had the following values (Supplementary Figure 3): on a biosecurity scale from 0 to 8, 50% of agents had initial biosecurity = 0 (no biosecurity); 40% of agents had initial biosecurity=2.7 (low), 10% had initial biosecurity=5.3 (medium) and 0% had initial biosecurity=8 (high). This initial model configuration of biosecurity represents a situation in which most producers implement biosecurity on a low-to-moderate level

before the simulated PEDv incursion. We made this assumption based on exchanges with veterinarian collaborators.

Supplementary Table 5. Calibration parameters, their search value range and incremental step and the parameter values on which the objective function converged. The last row reports the objective function score for the best parameter set.

**Parameter**	**Search range**	**Search step**	**Best score values**
Biosecurity increase	1.0 - 2.2	0.2	1.4
Psychological distancing	0.004 - 0.012	0.002	0.004
Delay of response to disease	0.08 - 0.12	0.01	0.1
Objective function (OF)			**3.04**

The calibration converged on the set of parameter values shown in Supplementary Table 4. The 136 OF scores ranged from 3.04 to 4.75. We decided to extract the parameter sets that had only slightly higher OF scores than the best fit (OF ≤ 3.19; ~ 50% of data, Supplementary Figure 5) to analyze the uniqueness of the best fit. We found a relationship among the parameter values of these sets meaning first that there are constraints among the human behavioral parameters necessary to fit the observed incidence data and second that a range of reasonably consistent human behavioral processes can give rise to the observed patterns. A Pearson correlation matrix (Supplementary Table 6), helps explain how these constraints interplay to create a decreasing trend in the PEDv incidence. If psychological distancing increases (higher relaxation rate in biosecurity), the disease incidence trend can only be negative when the producer agents respond to disease with shorter delay and higher biosecurity increase. If instead psychological distancing decreases, meaning that the level of biosecurity is generally maintained at all times, it is possible to reduce the output incidence even in the case when the agent producers act with some delay in their response to disease and they only increase biosecurity by little.

To choose the parameter values for our study, we took the 10 best OF solutions (OF score ≤ 3.09, 5% of data, Supplementary Figure 5) and selected the parameter set with intermediate values within the ranges covered by these best solutions. The scenario analysis described in this paper was therefore run with the following values: delay of response = 0.09; biosecurity increase = 1.4; psychological distancing = 0.008.

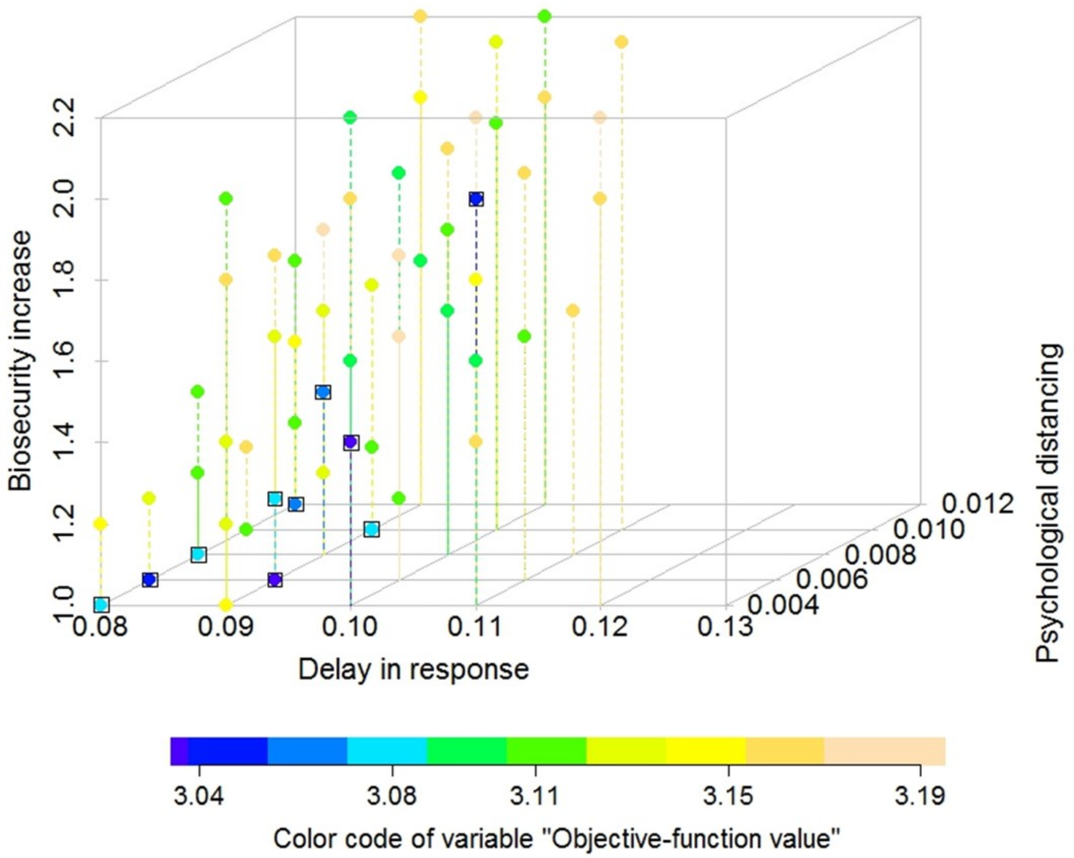

Supplementary Figure 5. Calibration parameter sets and related objective-function (OF) scores. The graph reports the subset (50%) of parameter sets with OF score ≤ 3.19. The three calibration variables – biosecurity increase, delay in response and psychological distancing – are represented by the three dimensions of the cube, while the variable OF is represented by color. The lower the OF score, the better is the fit to the observed data. The points of the 10 best parameter combinations are surrounded by a square symbol (OF score ≤ 3.09).

Supplementary Table 6. Pearson correlation values among the three human behavioral parameters varied in the calibration. The correlation values are for the subset of parameter sets with OF score < 3.19. Significance codes: 0 '***' 0.001 '**' 0.01 '*' 0.05 '.' 0.1 ' ' 1

	Biosecurity increase	Psychological distancing	Delay of response
Biosecurity increase	1.00		
Psychological distancing	0.037	1.00	
Delay of response	0.64 ***	-0.35 **	1.00

6 References

Wiltshire, S., Zia, A., Koliba, C., Bucini, G., Clark, E., Merrill, S., et al. (2019). Network Meta-Metrics: Using Evolutionary Computation to Identify Effective Indicators of Epidemiological Vulnerability in a Livestock Production System Model. *Journal of Artificial Societies and Social Simulation* 22(2)**,** 8. doi: 10.18564/jasss.3991.

Wiltshire, S.W. (2018). Using an agent-based model to evaluate the effect of producer specialization on the epidemiological resilience of livestock production networks. *PLOS ONE* 13(3)**,** e0194013. doi: 10.1371/journal.pone.0194013.
